# Supplementary material for: SARS-CoV-2 viral load is associated with increased disease severity and mortality
Source: Nat Commun. 2020 Oct 30;11:5493. doi: 10.1038/s41467-020-19057-5 (PMC7603483; doi:10.1038/s41467-020-19057-5)
Supplement: Supplementary file 1 — Supplementary Information [file 41467_2020_19057_MOESM1_ESM.pdf]

SARS-CoV-2 Viral Load is Associated with Increased Disease Severity and Mortality
Supplementary Figures and Tables

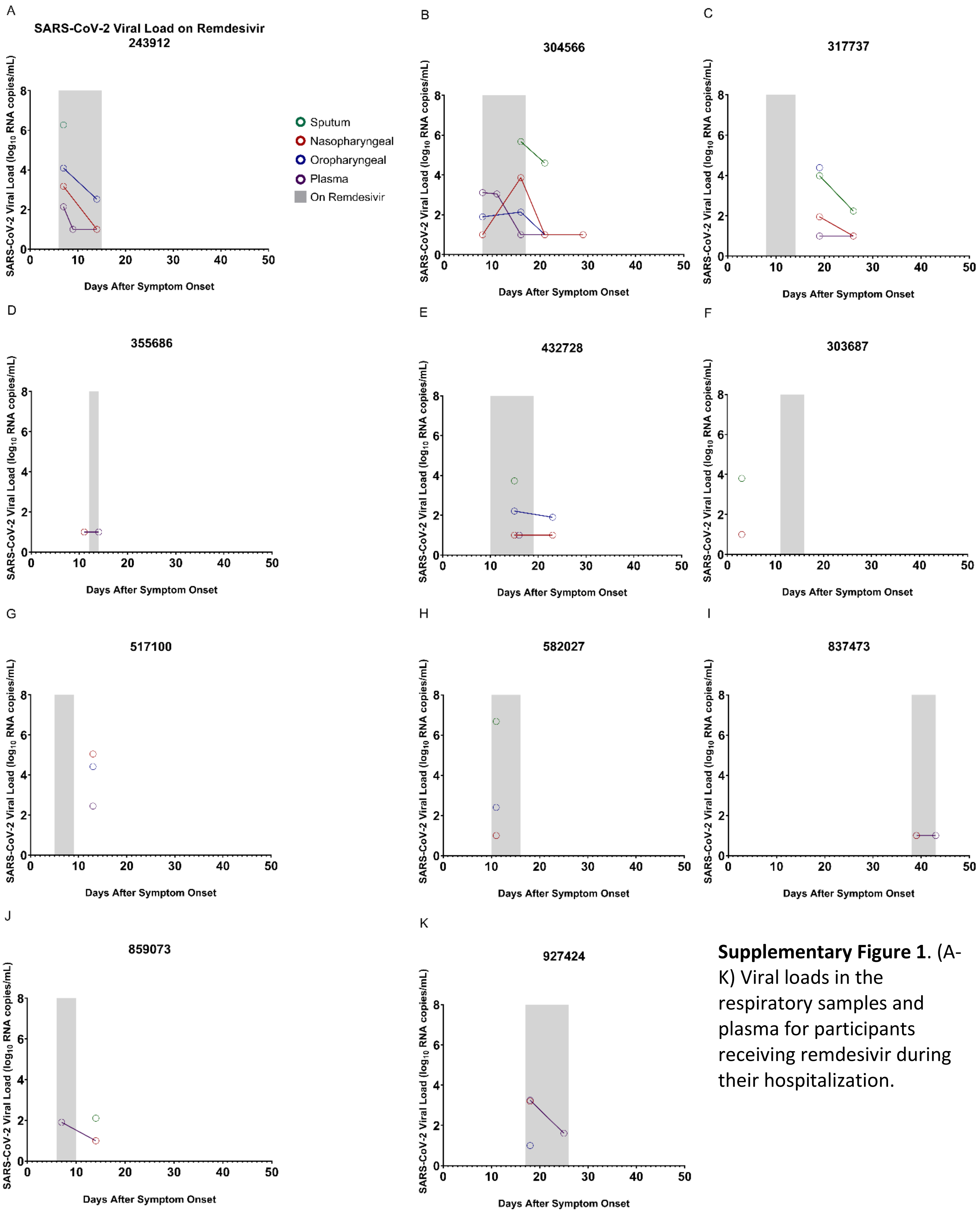

Supplementary Figure 1. (A-K) Viral loads in the respiratory samples and plasma for participants receiving remdesivir during their hospitalization.

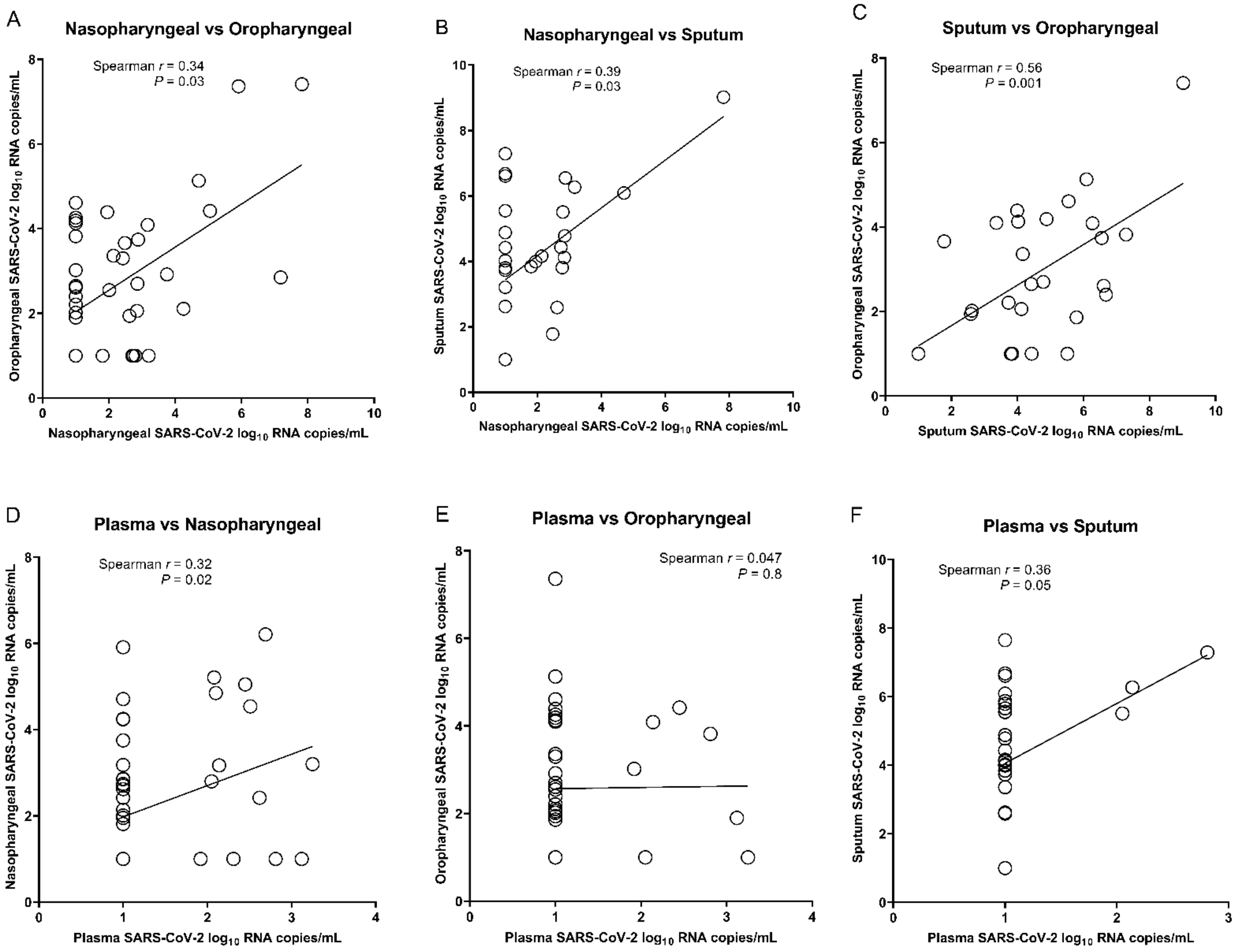

Supplementary Figure 2. Correlation of respiratory tract and plasma viral loads. (A) Nasopharyngeal vs Oropharyngeal, (B) Nasopharyngeal vs Sputum, (C) Sputum vs Oropharyngeal, (D) Plasma vs Nasopharyngeal, (E) Plasma vs Oropharyngeal, (F) Plasma vs Sputum. VL, viral load. P-values are from Spearman's correlations.

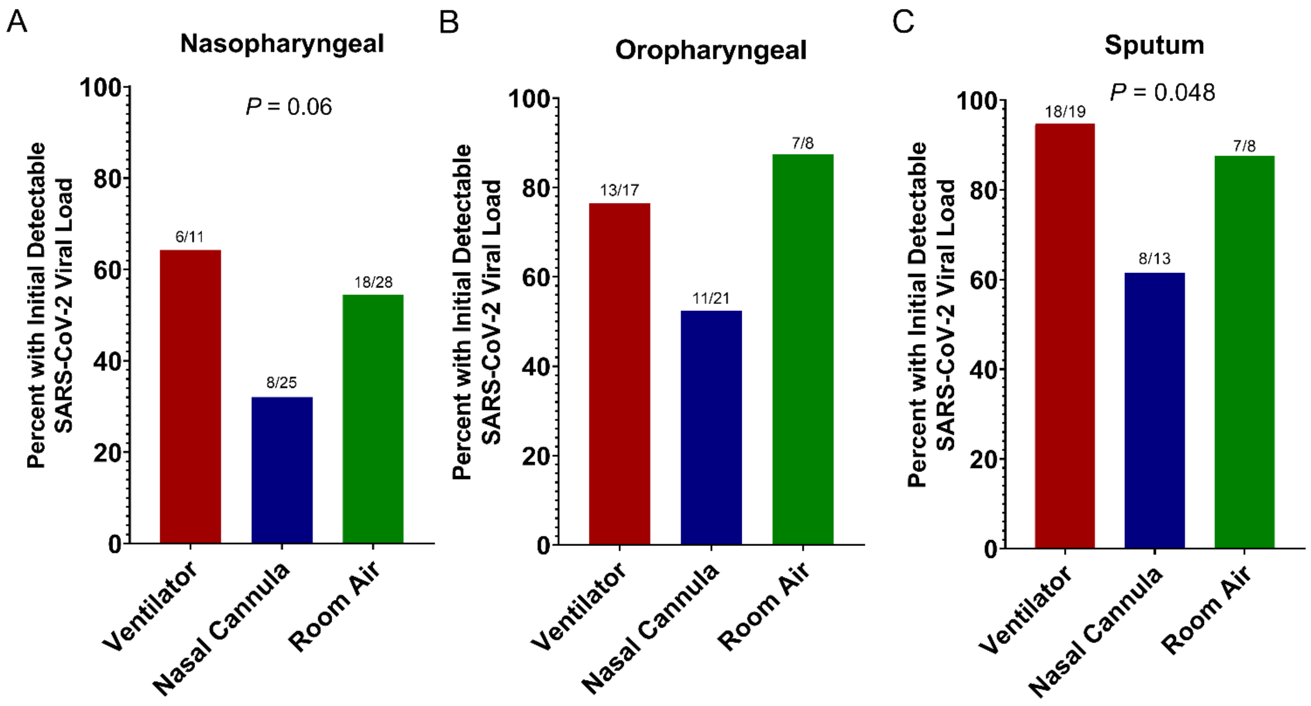

Supplementary Figure 3. Percentage of participants with a detectable SARS-CoV-2 viral load from nasopharyngeal swabs (A), oropharyngeal swabs (B), and sputum (C) collected at initial sampling. Data is further categorized by respiratory disease severity. P-values are from X^2 analysis.

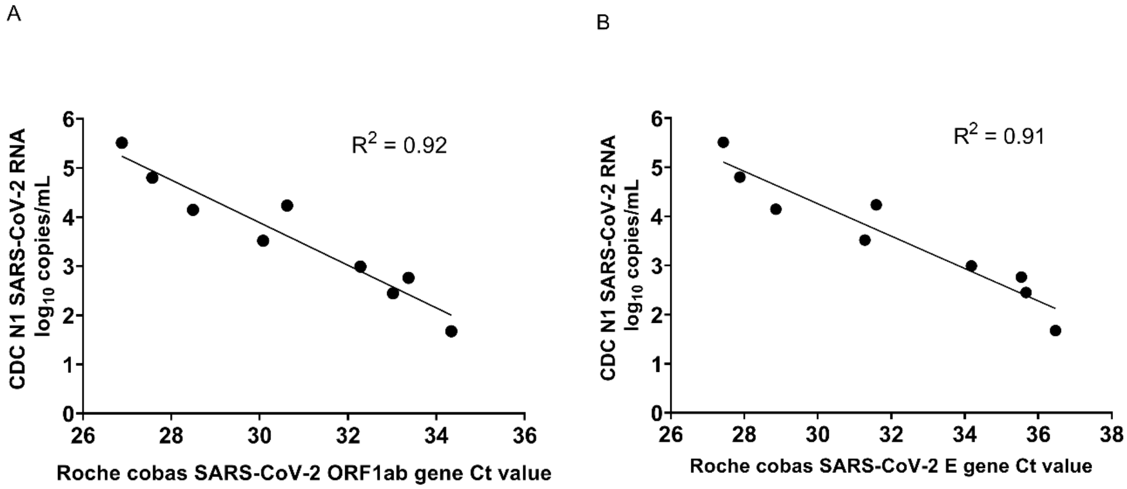

Supplementary Figure 4. Correlation of the CDC N1 SARS-CoV-2 viral load assay with the Roche cobas ORF1ab (A) and E (B) gene Ct (cycle threshold) values. P-values are from regression R^2.

Supplementary Table 1. Logistic regression analysis of association of viral load with risk of death

Specimen Type	Variable type[1]	Odds Ratio	P-value
Plasma	Categorical	5.5	**0.02**
Nasopharyngeal	Categorical	2.4	0.25
Oropharyngeal	Categorical	3.5	0.27
Sputum[2]	Categorical	-	-
Plasma	Continuous	2.4	**0.04**
Nasopharyngeal	Continuous	1.4	0.09
Oropharyngeal	Continuous	2.1	**0.02**
Sputum	Continuous	2.8	**0.048**

[1]Categorical refers to analysis of viral loads as detectable or not detectable
[2]There were no deaths in participants with undetectable sputum viral loads
[3]P-values have been bolded to highlight significance

Supplementary Table 2. Details of the participants who died. Primary causes of death as noted in the medical chart. Additional factors that may have contributed to death are also noted.

No.	Age	Sex	Plasma SARS-CoV-2 RNA positive	Significant past medical conditions	Primary causes of death	Secondary causes of death
**1**	80	M	No	Diabetes, coronary artery disease, hypertension	Respiratory failure	COVID-19, bacterial pneumonia, acute kidney injury
**2**	69	F	No	Diabetes, hypertension, obesity	Respiratory failure	COVID-19, bacterial pneumonia, acute kidney injury, septic shock
**3**	57	M	No	Asthma, obesity	Respiratory failure, cerebral edema and hemorrhage	COVID-19, septic shock
**4**	79	M	No	COPD, diabetes, hypertension	Respiratory failure	COVID-19, bacterial pneumonia, acute kidney injury, septic shock
**5**	69	F	Not available	Goiter	Respiratory failure	COVID-19, bacterial pneumonia, acute kidney injury, septic shock
**6**	84	M	Yes	Diabetes, cirrhosis	Respiratory failure	COVID-19, ileus, bacterial pneumonia, septic shock
**7**	85	M	Yes	Hypertension	Respiratory failure	COVID-19, septic shock
**8**	72	M	Yes	Obesity	Respiratory failure	COVID-19, bacterial pneumonia, acute kidney injury, septic shock
**9**	70	M	Yes	COPD, head and neck cancer	Respiratory failure	COVID-19, head and neck cancer, ileus, bacterial pneumonia, non-ST-elevation myocardial infarction, septic shock
**10**	76	M	Yes	Hypertension	Respiratory failure	COVID-19, bacterial pneumonia, acute kidney injury, possible pulmonary embolism, septic shock
**11**	79	M	Yes	Hypertension	Respiratory failure	COVID-19, acute kidney injury, septic shock, bacterial pneumonia, atrial fibrillation

SARS-CoV-2, severe acute respiratory syndrome coronavirus 2; COVID-19, coronavirus disease 19; COPD, chronic obstructive pulmonary disease

Supplementary Table 3. Complete list of primers.

Primer Name	Assay	Sequence (5' -> 3')
2019-nCoV_N1-F	SARS-CoV-2	GAC CCC AAA ATC AGC GAA AT
2019-nCoV_N1-R	SARS-CoV-2	TCT GGT TAC TGC CAG TTG AAT CTG
2019-nCoV_N1-P	SARS-CoV-2	FAM-ACC CCG CAT TAC GTT TGG TGG ACC-BHQ1
RCAS-F	RCAS	GTC AAT AGA GAG AGG GAT GGA CAA A
RCAS-R	RCAS	TCC ACA AGT GTA GCA GAG CCC
RCAS-P	RCAS	FAM- TGG GTC GGG TGG TCG TGC C-TAMRA
IPO8-F	IPO8	Biorad Unique Assay ID: qHsaCED0005354
IPO8-R	IPO8	Biorad Unique Assay ID: qHsaCED0005354
