# Supplementary material for: SARS-CoV-2 viral load is associated with increased disease severity and mortality
Source: Nat Commun. 2020 Oct 30;11:5493. doi: 10.1038/s41467-020-19057-5 (PMC7603483; doi:10.1038/s41467-020-19057-5)
Supplement: Supplementary file 2 — Reporting Summary [file 41467_2020_19057_MOESM2_ESM.pdf]

nature research

Corresponding author(s): Jonathan Li, MD

Last updated by author(s): Sep 7, 2020

# Reporting Summary

Nature Research wishes to improve the reproducibility of the work that we publish. This form provides structure for consistency and transparency in reporting. For further information on Nature Research policies, see our Editorial Policies and the Editorial Policy Checklist.

## Statistics

For all statistical analyses, confirm that the following items are present in the figure legend, table legend, main text, or Methods section.

| n/a | Confirmed | |
|---|---|---|
| ☐ | ☒ | The exact sample size (*n*) for each experimental group/condition, given as a discrete number and unit of measurement |
| ☐ | ☒ | A statement on whether measurements were taken from distinct samples or whether the same sample was measured repeatedly |
| ☐ | ☒ | The statistical test(s) used AND whether they are one- or two-sided<br>*Only common tests should be described solely by name; describe more complex techniques in the Methods section.* |
| ☐ | ☒ | A description of all covariates tested |
| ☒ | ☐ | A description of any assumptions or corrections, such as tests of normality and adjustment for multiple comparisons |
| ☐ | ☒ | A full description of the statistical parameters including central tendency (e.g. means) or other basic estimates (e.g. regression coefficient) AND variation (e.g. standard deviation) or associated estimates of uncertainty (e.g. confidence intervals) |
| ☐ | ☒ | For null hypothesis testing, the test statistic (e.g. *F*, *t*, *r*) with confidence intervals, effect sizes, degrees of freedom and *P* value noted<br>*Give P values as exact values whenever suitable.* |
| ☒ | ☐ | For Bayesian analysis, information on the choice of priors and Markov chain Monte Carlo settings |
| ☒ | ☐ | For hierarchical and complex designs, identification of the appropriate level for tests and full reporting of outcomes |
| ☒ | ☐ | Estimates of effect sizes (e.g. Cohen's *d*, Pearson's *r*), indicating how they were calculated |

*Our web collection on statistics for biologists contains articles on many of the points above.*

## Software and code

Policy information about availability of computer code

| | |
|---|---|
| Data collection | No software was used. |
| Data analysis | Logistic regression and other statistical analyses were performed using GraphPad Prism 8 and SAS software, version 9. |

For manuscripts utilizing custom algorithms or software that are central to the research but not yet described in published literature, software must be made available to editors and reviewers. We strongly encourage code deposition in a community repository (e.g. GitHub). See the Nature Research guidelines for submitting code & software for further information.

## Data

Policy information about availability of data

All manuscripts must include a data availability statement. This statement should provide the following information, where applicable:

- Accession codes, unique identifiers, or web links for publicly available datasets
- A list of figures that have associated raw data
- A description of any restrictions on data availability

The authors declare that the data supporting the findings of this study are available within the article and its supplementary data files.

# Field-specific reporting

# Life sciences study design

All studies must disclose on these points even when the disclosure is negative.

| | |
|---|---|
| Sample size | We enrolled a convenience sample of hospitalized and non-hospitalized participants with COVID-19 who agreed to participate in a prospectively-collected longitudinal sample collection study conducted at the Massachusetts General Hospital and the Brigham and Women's Hospital. |
| Data exclusions | No data was excluded from our study. |
| Replication | The results are concordant between multiple sample types and in multiple subgroup analysis. |
| Randomization | This was an observational study and therefore no randomization was used. |
| Blinding | There was no intervention or blinding as part of this study. |

# Reporting for specific materials, systems and methods

We require information from authors about some types of materials, experimental systems and methods used in many studies. Here, indicate whether each material, system or method listed is relevant to your study. If you are not sure if a list item applies to your research, read the appropriate section before selecting a response.

## Materials & experimental systems

| n/a | Involved in the study | |
|---|---|---|
| ☒ | ☐ | Antibodies |
| ☒ | ☐ | Eukaryotic cell lines |
| ☒ | ☐ | Palaeontology and archaeology |
| ☒ | ☐ | Animals and other organisms |
| ☐ | ☒ | Human research participants |
| ☒ | ☐ | Clinical data |
| ☒ | ☐ | Dual use research of concern |

## Methods

| n/a | Involved in the study | |
|---|---|---|
| ☒ | ☐ | ChIP-seq |
| ☒ | ☐ | Flow cytometry |
| ☒ | ☐ | MRI-based neuroimaging |

## Human research participants

Policy information about studies involving human research participants

| | |
|---|---|
| Population characteristics | Demographics information can be found in Table 1 and additional information on antiviral treatment can be found in Supplemental Figure 1. Only univariate analysis was performed due to the available sample size, but we did perform a sensitivity analysis for plasma viral load effects based on disease severity and age. |
| Recruitment | We enrolled hospitalized and non-hospitalized participants with COVID-19 in a longitudinal sample collection study at two academic medical centers. The population of recovered individuals who agreed to participate in this study were younger and had fewer comorbidities compared to hospitalized participants, potentially affecting the generalizability of the results in the cohort of individuals with recovered COVID-19 disease. |
| Ethics oversight | This study was approved by the Partners Institutional Review Board. |

Note that full information on the approval of the study protocol must also be provided in the manuscript.
